# The Genome of *Vitis zhejiang-adstricta* Strengthens the Protection and Utilization of the Endangered Ancient Grape Endemic to China

**DOI:** 10.1093/pcp/pcad140

**Published:** 2023-11-01

**Authors:** Huayang Li, Yongbo Liu, Peige Fan, Zhanwu Dai, Jiachen Hao, Wei Duan, Zhenchang Liang, Yi Wang

**Affiliations:** Beijing Key Laboratory of Grape Science and Enology, CAS Key Laboratory of Plant Resources, Institute of Botany, Chinese Academy of Sciences, 20 Nanxincun, Xiangshan, Beijing 100093, China; State Key Laboratory of Plant Diversity and Specialty Crops, Institute of Botany, Chinese Academy of Sciences, 20 Nanxincun, Xiangshan, Beijing 100093, PR China; China National Botanical Garden, 20 Nanxincun, Xiangshan, Beijing 100093, PR China; University of Chinese Academy of Sciences, 19 Yuquan Rd, Beijing 100049, PR China; State Key Laboratory of Environmental Criteria and Risk Assessment, Chinese Research Academy of Environmental Sciences, 8 Dayangfang, Beijing 100012, PR China; Beijing Key Laboratory of Grape Science and Enology, CAS Key Laboratory of Plant Resources, Institute of Botany, Chinese Academy of Sciences, 20 Nanxincun, Xiangshan, Beijing 100093, China; State Key Laboratory of Plant Diversity and Specialty Crops, Institute of Botany, Chinese Academy of Sciences, 20 Nanxincun, Xiangshan, Beijing 100093, PR China; China National Botanical Garden, 20 Nanxincun, Xiangshan, Beijing 100093, PR China; Beijing Key Laboratory of Grape Science and Enology, CAS Key Laboratory of Plant Resources, Institute of Botany, Chinese Academy of Sciences, 20 Nanxincun, Xiangshan, Beijing 100093, China; State Key Laboratory of Plant Diversity and Specialty Crops, Institute of Botany, Chinese Academy of Sciences, 20 Nanxincun, Xiangshan, Beijing 100093, PR China; China National Botanical Garden, 20 Nanxincun, Xiangshan, Beijing 100093, PR China; China National Botanical Garden, 20 Nanxincun, Xiangshan, Beijing 100093, PR China; Beijing Key Laboratory of Grape Science and Enology, CAS Key Laboratory of Plant Resources, Institute of Botany, Chinese Academy of Sciences, 20 Nanxincun, Xiangshan, Beijing 100093, China; State Key Laboratory of Plant Diversity and Specialty Crops, Institute of Botany, Chinese Academy of Sciences, 20 Nanxincun, Xiangshan, Beijing 100093, PR China; China National Botanical Garden, 20 Nanxincun, Xiangshan, Beijing 100093, PR China; Beijing Key Laboratory of Grape Science and Enology, CAS Key Laboratory of Plant Resources, Institute of Botany, Chinese Academy of Sciences, 20 Nanxincun, Xiangshan, Beijing 100093, China; State Key Laboratory of Plant Diversity and Specialty Crops, Institute of Botany, Chinese Academy of Sciences, 20 Nanxincun, Xiangshan, Beijing 100093, PR China; China National Botanical Garden, 20 Nanxincun, Xiangshan, Beijing 100093, PR China; Beijing Key Laboratory of Grape Science and Enology, CAS Key Laboratory of Plant Resources, Institute of Botany, Chinese Academy of Sciences, 20 Nanxincun, Xiangshan, Beijing 100093, China; State Key Laboratory of Plant Diversity and Specialty Crops, Institute of Botany, Chinese Academy of Sciences, 20 Nanxincun, Xiangshan, Beijing 100093, PR China; China National Botanical Garden, 20 Nanxincun, Xiangshan, Beijing 100093, PR China

**Keywords:** Disease resistance, Evolution, Expanded and contracted gene family, Genome, *V. zhejiang-adstricta*

## Abstract

*Vitis zhejiang-adstricta* (*V. zhejiang-adstricta*) is one of the most important and endangered wild grapes. It is a national key protected wild, rare and endangered ancient grape endemic to China and used as a candidate material for resistance breeding owing to its excellent significant disease resistance. Here, we present a high-quality chromosome-level assembly of *V. zhejiang-adstricta* (IB-VB-01), comprising 506.66 Mb assembled into 19 pseudo-chromosomes. The contig N50 length is 3.91 Mb with 31,196 annotated protein-coding genes. Comparative genome and evolutionary analyses illustrated that *V. zhejiang-adstricta* has a specific position in the evolution of East Asian *Vitis* and shared a common ancestor with *Vitis vinifera* during the divergence of the two species about 10.42 (between 9.34 and 11.12) Mya. The expanded gene families compared with those in plants were related to disease resistance, and constructed gene families were related to plant growth and primary metabolism. With the analysis of gene family expansion and contraction, the evolution of environmental adaptability and especially the NBS-LRR gene family of *V. zhejiang-adstricta* was elucidated based on the pathways of resistance genes (R genes), unique genes and structural variations. The near-complete and accurate diploid *V. zhejiang-adstricta* reference genome obtained herein serves as an important complement to wild grape genomes and will provide valuable genomic resources for investigating the genomic architecture of *V. zhejiang-adstricta* as well as for improving disease resistance breeding strategies in grape.

## Introduction

*V. zhejiang-adstricta* is a national key protected wild, rare and endangered ancient grape endemic to China, which is only distributed in a few mountainous places in Zhejiang Province, China (Wan et al. 2008). As a rare local variety of *V. adstricta* (Kong 2004, He 2012), *V. zhejiang-adstricta* was listed as a second-level protected plant in the National Key Protected Wild Plant List released in 2021. The population of *V. zhejiang-adstricta* is not easily reproduced by seeds, and its habitats are restricted and easily altered by human activities (Jiang et al. 2015, Min et al. 2018). The investigation of these species revealed that *V. zhejiang-adstricta* has difficult fruit setting, reproduces slowly and has a slow population recovery. However, *V. zhejiang-adstricta* can live in humid native habitats attributed to its remarkable disease resistance (Wan et al. 2008). Therefore, the protection and utilization of *V. zhejiang-adstricta* should establish a novel and valuable foundation, which may contribute to the understanding of grape breeding applications.

With the development of sequencing technology, the genome sequences of numerous *Vitis vinifera* varieties including Pinot Noir PN40024 (Jaillon et al. 2007), Cabernet (Chin et al. 2016) and Chardonnay (Roach et al. 2018a) have been released. In contrast to *V. vinifera*, fewer studies have been conducted on wild *Vitis* species owing to the assembly difficulties caused by high heterozygosity (Chin et al. 2016). According to previous research statistics, 13 wild grape genomes have been released, including two Chinese wild grape varieties (Wang et al. 2021, Velt et al. 2023, Dong et al. 2023a). Nonetheless, wild *Vitis* species have several beneficial traits, such as fruit quality–related attributes and resilience to biotic and abiotic stresses (Jiang et al. 2015). With the development of sequencing strategies, high-throughput chromosome conformation capture (Hi-C) and long reads of single molecular sequencing technologies have made the genome assembly of these species easier to implement and 12 wild grapes which have been published (Dong et al. 2023a; Wang et al. 2021, Noé et al. 2023) have revealed the genetic basis of the adaptive traits, potentially accelerating grape breeding research.

Throughout the species evolution process, most plants experienced one or more ancient genome polyploidization. Whole-genome duplication (WGD) is deemed to contribute greatly to speciation and the emergence of many valuable traits in plants (Rensing 2014). The genome sequence project of grapevine suggested that grape only experienced the ancestral hexaploidization out of the major angiosperm phyla at 120∼150  million years ago (Mya) (Jaillon et al. 2007) and no massive segment duplication or chromosome-level variation occurred after this event. Hence, grape was considered to be one of the most ancient angiosperms (Jaillon et al. 2007). In addition, *Vitis* or even Vitaceae plants were utilized widely in the evolution analysis for their close relationship to the common ancestor of the angiosperm (Xin et al. 2022). Hexaploidization produces dissimilar subgenomes, and genes are unequally lost from subgenomes. WGD events initially not only double the genome but also gain and lose gene copies in those events (Van de Peer et al. 2009). Previous studies have identified useful genes derived from WGDs that are related to plant growth and metabolic pathways (Chae et al. 2014, Chakraborty 2018).

In this study, we aimed to assemble a high-quality chromosome-scale genome of *V. zhejiang-adstricta* using Nanopore long reads, Illumina reads and Hi-C data. We found lineage-specific expansion and contraction of gene families, which may contribute to exploring the origin and environment adaption mechanism of *V. zhejiang-adstricta*. The high-quality genome of *V. zhejiang-adstricta* provides new insights into the molecular basis of disease resistance pathways and may provide benefits to grape breeding programs.

## Results

### Sequencing and assembly of a high-quality loquat draft genome

**Fig. 1:**
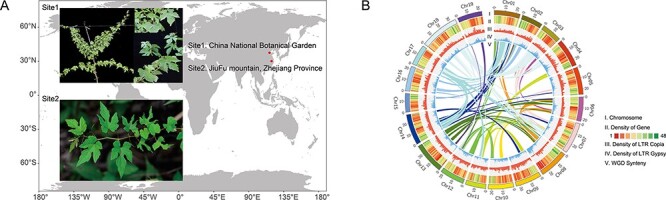
Phenotypic characteristics and genome information of *V. zhejiang-adstricta*. (A) Location of *V. zhejiang-adstricta* and its tissues (leaf, fruit) used in this study, Site 1. *V. zhejiang-adstricta* expanding propagation in the vineyard of China National Botanical Garden, Site 2. *V. zhejiang-adstricta* sampling in the JiuFu mountain nature reserve. (B) Synteny, gene and LTR distribution of the *V. zhejiang-adstricta* genome. As indicated in the inset, the rings indicate (from outside to inside) (I) chromosomes, (II) heat maps representing gene density, (III) Gypsy-type TE density, (IV) Copia-type TE density and (V) homologous regions of the pear genome, which are connected by lines representing syntenic regions identified by MCScanX and mapped using Circos software.

*V. zhejiang-adstricta* (2*n* = 2*x* = 38) is an endangered species that only exists in a small area of Zhejiang province, China (**Fig. 1A**). In this study, 32.88-Gb clean short reads were used to survey the genome size and heterozygosity of this genome. We found that the genome size of *V. zhejiang-adstricta* was ∼510 Mb and the heterozygosity rate was 0.845% based on the K-mer analysis (K-mer = 25) (**Supplementary Fig. S1**).

Whole-genome sequencing was then performed, and we obtained about 98.51 Gb (∼194-fold coverage) Nanopore data (**Supplementary Table S1**); after correcting and trimming by CANU, 24.28-Gb (∼47.61-fold coverage) high-quality data (total number of subreads = 428,721 reads, subread N50 = 60 kb) remained. With these high-quality subreads, an initial draft genome composed of 2,219 contigs was generated with a genome size of 521.76 Mb and a contig N50 of 3.91 Mb. Assisted by the Hi-C data, a total of 19 pseudo-chromosome genomes contain 506.66 Mb and an N50 of ∼23.08 Mb, covering ∼99.35% of the estimated genome size (**Fig. 1B**, **Supplementary Fig. S2**).

Three methods were adopted to assess the completeness of the draft genome sequences. First, the clean WGS datasets were mapped onto the assembly, and the result indicated that 95.20% of the draft genome could be aligned properly (**Table 1**). Second, Benchmarking Universal Single-Copy Orthologs (BUSCO) analysis indicated that 1,950 genes (92%) could be found in the plant core genes/orthologs. Meanwhile, the result of LTR Assembly Index (LAI) showed that the LAI index of *V. zhejiang-adstricta* is 16, which means that this assembly belongs to the category of a reference genome. All these results illustrated that the *V. zhejiang-adstricta* genome is of high quality and could be employed as a reference whole genome.

**Table 1:** Summary statistics of the assembly and annotation of the *V. zhejiang-adstricta* genome

Assembly v1.0 (chromosome level)	
Number of chromosomes	2*n* = 38
Estimated genome size (Mb)	∼510
Number of scaffolds	2,219
Total assembled genome (Mb)	507
Longest scaffold (Mb)	14.91
Scaffolds N50 (Mb)	3.82
Scaffolds L50	36
Gaps (Mb)	2.2
Number of pseudo-chromosomes	19
GC content	34.62%
Embryophyta 1614 BUSCO	92%
LAI	16
Annotation and validation	
Number of gene models	31,196
Number of exons per gene	4.77
Repetitive sequence size (Mb)	240
Genes annotated with public databases	93.71%
Noncoding RNAs	1,289

### Genome model annotation of *V. zhejiang-adstricta*

The identified repetitive sequence size was 240.62 Mb, which masked 47.49% of the *V. zhejiang-adstricta* genome size. LTR accounted for 29.50% of the genome, and DNA elements accounted for 8.15% of the genome (**Supplementary Table S2**). The long interspersed nuclear elements (LINEs) and short interspersed nuclear elements occupied 4.31% of the whole-genome sequences. All satellites only accounted for 0.01% of the genome. Many plant genomes are composed of a large number of transposable elements, with the published *Vitis* reference genome transposable elements accounting for 44.91% of *V. vinifera* and 47.06% of *V. amurensis*. In *V. zhejiang-adstricta*, we identified 47.49% of the diploid genome as repetitive sequences. Across all three species, similar distributions of the repetitive classes were also observed. LTR elements including Copia and Gypsy were the most abundant repetitive elements in all four sequenced genomes, and LINEs were the main proportion of non-LTR elements (**Supplementary Table S2**).

A combined method involving *ab initio* gene prediction, homology-based searches and transcriptome evidence was employed to predict the coding genes in *V. zhejiang-adstricta* genome. A total of 31,196 protein-coding genes were identified in the *V. zhejiang-adstricta* genome. The average gene length was 4,685 bp, and the average exon length was 1,172 bp (**Table 1**). Furthermore, ∼93.71% of the predicted protein-coding genes of *V. zhejiang-adstricta* could be functionally annotated by Nr, Pfam, Uniprot, GO and KEGG databases (**Table 1**). In addition, 1,588 TFs (transcription factors) were identified in the *V. zhejiang-adstricta* genome, which could be divided into 58 TF gene families, including 160, 122, 114 and 87 members of MYB, bHLH, ERF and NAC, respectively(**Supplementary Table S3**). Besides these protein-coding genes, 785 tRNAs, 617 ribosomal RNAs (rRNAs), 492 miRNAs and 391 snRNAs were also identified in this genome (**Supplementary Fig. S3**).

### Genome evolution and comparative genomic analysis

**Fig. 2:**
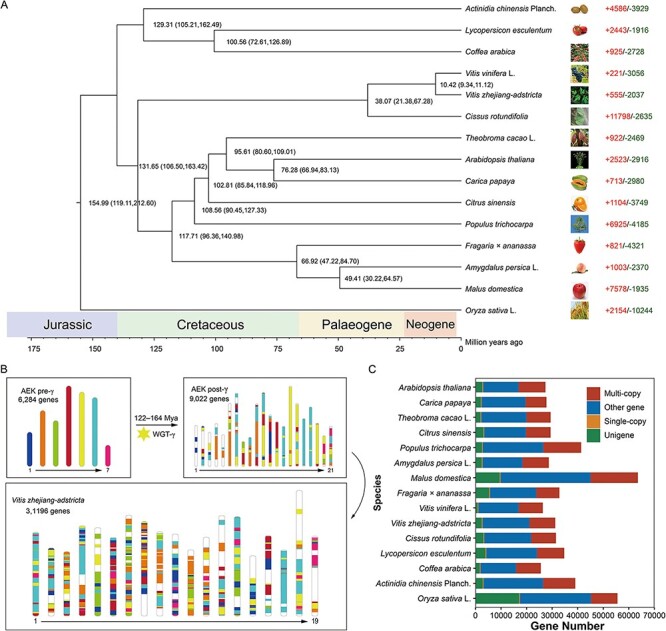
Evolution history and gene family clustering for 15 species including *V. zhejiang-adstricta* and chromosomal rearrangement of *V. zhejiang-adstricta* against AEK and AEK-γ. (A). Phylogenetic tree and divergence times of *V. zhejiang-adstricta* and 15 species based on 307 single-copy genes, and the positive and negative numbers represent expanded and contracted gene families, respectively. (B). Reconstruction of ancestral chromosomes of *V. zhejiang-adstricta* compared with AEK recent common ancestor chromosomes after the WGD event. (C) The shared and unique gene families in 15 plants.

The origin and divergence times of *V. zhejiang-adstricta* were measured by comparative genomic analysis with another 14 representative plants. In total, 306 single-copy genes shared by all 15 species were used to construct the phylogenetic tree. With the reference divergence time of *Arabidopsis thaliana* and *Oryza sativa* L. or *Carica papaya*, the phylogenetic tree inferred that *V. zhejiang-adstricta* shared a common ancestor with *V. vinifera*, ∼9.34–11.12 Mya and divergence of these species occurred at ∼10.42 Mya. *Vitis* as predicted shared a common ancestor with *Cissus rotundifolia*, about ∼38.07 Mya (21.38–67.26 Mya) (**Fig. 2A**).

WGD analysis was conducted to explore the genome duplication events. In total, 2,210 segment duplication events were identified in the *V. zhejiang-adstricta* genome, and the total length of those segments accounted for 38.64% of the genome. Besides these segment duplications, 3,788 tandem duplication events were also identified in the *V. zhejiang-adstricta* genome, and only 2,757 in *V. vinifera*. Palaeohistory analysis of *V. zhejiang-adstricta* was according to the method of reconstructed ancestral eudicot karyotype (AEK) genes. The total number of identified orthologous genes between *V. zhejiang-adstricta* and AEK pre-WGT-γ was 20,252, accounting for 58.04% region of the total genome. Those orthologous regions accounted for 48.19% between *V. vinifera* and AEK pre-WGT-γ. In addition to the AEK pre-WGT-γ, the orthologous genes between *V. zhejiang-adstricta* and AEK post-WGT-γ were also identified using those methods. The total number of orthologous genes was 21,935, accounting for 65.86% of the *V. zhejiang-adstricta* genome (**Fig. 2B**). Although many structure variations also occurred during its evolution, all the proto-chromosomes of AEK and AEK-γ events could be found in the corresponding syntenic blocks in *V. zhejiang-adstricta*.

The synonymous nucleotide substitution (Ks) abundance of *V. zhejiang-adstricta* and *V. vinifera* L. indicated that they shared the same WGD events at 2.35, which occurred at 122–164 Mya (**Fig. 2A**, **4B**). Furthermore, *V. zhejiang-adstricta* and *V. vinifera* have a divergence peak at 0.05, indicating that the divergence time of *V. zhejiang-adstricta* and *V. vinifera* could be traced back to 9.09–11.78 Mya; this value was similar to the MCMC result (**Fig. 2A**). Furthermore, comparative and evolutionary histories of orthologous genes were identified based on the gene cluster of *V. zhejiang-adstricta* and another 15 plants. In this study, 34,473 gene families were obtained. Among these families, all 31,196 genes from *V. zhejiang-adstricta* were aligned into 14,903 gene families (**Fig. 2C**). This result was consistent with a previous study in *V. amurensis*. In addition, 2,460 specific genes were identified in *V. zhejiang-adstricta*; these specific genes had a 2568 GO function and were enriched in 149 GO terms (**Supplementary Fig. S4**). The significant terms of gene functions include binding, catalytic activity, cellular process, response to stimulus, response to stress, etc. According to the KEGG pathway analysis, those were enriched in plant–pathogen interactions, MAPK signaling pathway and betalain biosynthesis. The function of specific genes was mainly related to response stress and growth of *V. zhejiang-adstricta*.

Besides the specific genes, *V. zhejiang-adstricta* also had 81 families consisting of 555 expanded genes (**Supplementary Table S4**) and 28 families consisting of 2,037 contracted genes (**Supplementary Table S5**). *Vitis vinifera* have more contraction and less expansion than *V. zhejiang-adstricta*, which means that *V. vinifera* may have lost more genetic diversity during the long period of species formation. The GO terms of contracted genes were enriched into cell homeostasis, protein modification process, primary metabolic process, cell morphogenesis, growth, enzymatic activity, binding, etc (**Supplementary Fig. S5**). The GO terms of expanded genes enriched into the secondary metabolic process, defense response, apoptosis, laccase activity, oxidoreductase activity, cell composition, etc (**Supplementary Fig. S5**). Most expanded gene families are related to plant irritability response and stress resistance, such as retrovirus-related pol poly protein, RPS, FAR, KIN and STS gene family. Most of the expanded gene families are related to plant irritability response, but most of the contracted gene families are related to plant growth and development.

### SVs and syntenic gene pairs between *V. zhejiang-adstricta* and the other two *Vitis* species

**Fig. 3:**
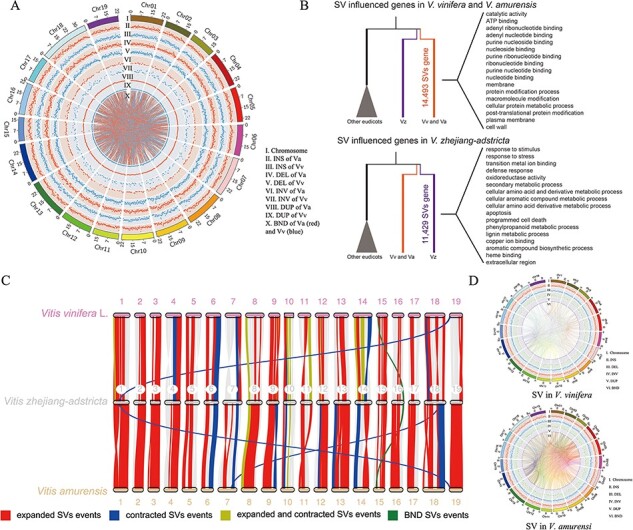
SV information and macro-synteny patterns between *V. zhejiang-adstricta* and *V. vinifera* or *V. amurensis*. (A) SV distribution of the *V. zhejiang-adstricta* genome against *V. vinifera*. As indicated in the inset, the rings indicate (from outside to inside) chromosomes (Chr), INS, DEL, INV and DUP. Inside the figure, homologous regions of the pear genome are connected by lines representing BND identified using Circos software. (B) SV distribution of the *V. zhejiang-adstricta* genome against with *V. amurensis*. As indicated in the inset, the rings indicate (from outside to inside) chromosomes (Chr), INS, DEL, INV and DUP. Inside the figure, homologous regions of the pear genome are connected by lines representing BND identified using Circos software. (C) Syntenic gene pairs of *V. zhejiang-adstricta* compared with other Vitis. (D) SV information of *V. zhejiang-adstricta* in *V. vinifera* or *V. amurensis.*

To deeply survey the landscape of natural structural variation (SV) between *V. zhejiang-adstricta* and other two *Vitis*, the long-corrected reads of *V. vinifera* and *V. amurensis* were mapped to *V. zhejiang-adstricta*, and a total of 197,304 and 162,045 SVs were identified from *V. amurensis* and *V. vinifera* long-reads mapping to *V. zhejiang-adstricta*, respectively (**Supplementary Table S6**, **Fig. 3A**). The SVs were assigned to five categories according to the reference; among these five types of SVs, insertions (INS) was the most common SV type (79,655 of *V. amurensis* and 70,714 of *V. vinifera*), followed by deletions (DEL) (68,906 of *V. amurensis* and 56,319 of *V. vinifera*), translocation breakpoints (BND) (41,182 of *V. amurensis* and 30,180 of *V. vinifera*), duplications (DUP) (6,011 of *V. amurensis* and 3,969 of *V. vinifera*) and inversions (INV) (1,550 of *V. amurensis* and 863 of *V. vinifera*). The density of SVs of *V. amurensis* and *V. vinifera* in the *V. zhejiang-adstricta* genome was 3.89 and 3.20 per 10kb, respectively. The distribution of these SVs was not uniform for all chromosomes, and chromosome 7 contained the most variations. According to the annotation, the most SVs were distributed in the intergenic regions, and 11,321 and 11,760 genes were affected by SVs in *V. vinifera* and *V. amurensis* referred to the *V. zhejiang-adstricta* genome. The affected genes of SVs take part in disease resistance and accelerate germination in *Vitis*. The affected genes of SVs including 40 genes (63.5%) experienced constriction events, and 548 genes (40.1%) experienced expansion events. The large segment variation genes are related to basal metabolism such as photosynthesis, cell death and cell cycle. Those gene related to DEL and DUP are mainly involved in defense and stress on ground of response to stress, response to stimulus and biotic stimuli, etc (**Fig. 3B**). In addition, we found a special BND event (chromosome level) that involves gene loss and gain. This BND variation contained three protein-coding genes that only existed in *V. zhejiang-adstricta*. Among them, VBG0100640 and VBG0100637 were related to disease resistance and VBG0127262 as a PELPK1-like gene was identified, which may accelerate germination and response to pathogens. The fragment position of this BND variant in *V. vinifera* and *V. amurensis* is relatively stable (**Fig. 3C**), and this BND variant was a unique variant type of *V. zhejiang-adstricta*.

To analyze the syntenic gene pairs of the *V. zhejiang-adstricta* genome against *V. vinifera* and *V. amurensis*, 640 syntenic gene pairs (containing 18,535 genes) were identified from *V. zhejiang-adstricta* and *V. vinifera* and 511 syntenic gene pairs (containing 18,842 genes) were identified from *V. zhejiang-adstricta* and *V. amurensis* (**Fig. 3C**, **D**). Syntenic gene pairs of *Vitis* parsed the relationship distance, the characteristics and independent evolution of *V. zhejiang-adstricta*.

### *V.zhejiang-adstricta* in the *Vitis* genus

**Fig. 4:**
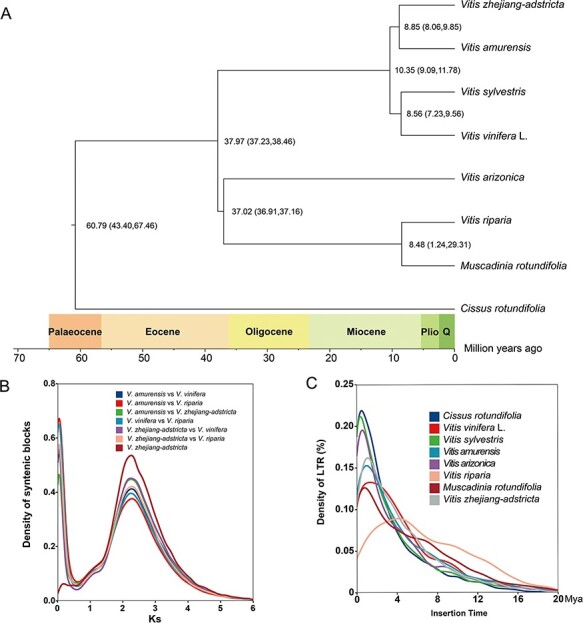
Evolution history of Vitis. (A) Phylogenetic tree and divergence times of *V. zhejiang-adstricta* and the other Vitis. (B) Synonymous nucleotide substitution (Ks) abundance distribution in *Vitis*. (C) Distribution of insertion times (Mya) for LTR-RTs in *Vitis.*

To obtain the characteristics and evolution of *V. zhejiang-adstricta* in the *Vitis* genus, 246 single-copy genes identified from the *Vitis* genus, including *C. rotundifolia* as an outgroup, were used to reconstruct the phylogenetic relationship tree (**Fig. 4A**). The phylogenic relationship tree illustrated that *V. zhejiang-adstricta* and *V. amurensis* diverged about 8.85 Mya (8.06–9.85 Mya), and the common ancestor of *V. zhejiang-adstricta* and *V. amurensis* diverged from the *V. vinifera* and *V. vinifera* sylvestris common ancestor about 10.35 Mya (9.09–11.78 Mya). *Vitis vinifera* shared a common ancestor with *V. vinifera* sylvestris, the divergence of which was about 8.56 Mya (7.23–9.56 Mya) (**Fig. 4B**, **Supplementary Table S7**). Particularly, the divergence time of *V. zhejiang-adstricta* in *Vitis* was earlier than that of *V. vinifera* and later than that of the American species. This result indicated that the origin and classification of Chinese wild *Vitis* had independent adaptive evolution against *V. vinifera* and the American species. In total, 410 *V. zhejiang-adstricta* specific genes were identified, and GO annotations of these genes indicated that they were enriched in 40 GO terms, including enzyme regulator activity, programmed cell death and defense response. Functional analysis indicated that these 119 genes include pathogenesis-related protein, disease resistance protein and Hsp90 protein. These genes retained after the divergence with other *Vitis* may contribute to the adaptation of *V. zhejiang-adstricta* to special environmental stress factors. When comparing leaf length and width, petiole length and internode length and width of 11 Chinese wild grapes and 2* V. vinifera*, the growth potential, especially leaf size and internode development, of *V. zhejiang-adstricta* is smaller than other Chinese wild grapes (**Supplementary Fig. S6**). Indeed, data from the phylogenetic tree analyses of Chinese wild grape implied that the speciation of *V. zhejiang-adstricta* was earlier than that of other *Vitis. V. zhejiang-adstricta, V. adstricta* and *Vitis pseudoreticulata* were found to have similar genetic distances in the phylogenetic tree, especially the relationship between *V. zhejiang-adstricta* and *V. adstricta*. Phylogenetic relationship accessions are grouped into other *Vitis* clades of different genetic backgrounds; among the wild grape varieties in China, the same kinds of grapes show aggregation (**Supplementary Fig. S7**).

Long terminal repeats-retrotransposons (LTR-RTs) were identified, and the insertion times of LTR-RTs estimated in *Vitis* illustrated that the insert events of large-scale LTR-RTs mainly affected plant genome size expansion and were restricted by the natural selection pressure (**Fig. 4C**). Insertion time analysis illustrated that most of them occurred recently in *V. zhejiang-adstricta*, explaining the complexity of these regions during the assembly process. The R-gene family is a significant group of genes that contain a nucleotide-binding site (NBS) domain and a leucine-rich repeat (LRR) domain and plays a crucial role in plant disease resistance (**Supplementary Fig. S8**). In the *V. zhejiang-adstricta* genome, 2,116 R genes were identified. Among the type CC, NBS, CN and CNL R genes of *V. zhejiang-adstricta* and *Muscadinia rotundifolia* have the highest percentage of the total genes in *Vitis*. The number of R genes identified in the *Vitis* was discrepant, and the number of R genes in *V. zhejiang-adstricta* was more than those in *V. vinifera* and *V. amurensis* (**Supplementary Table S8**).

## Discussion

### The high-quality genome of *V. zhejiang-adstricta* provides important genetic resources for the conservation of endangered species

In this study, a chromosome-level high-quality *V. zhejiang-adstricta* genome was presented with a genome size of ∼506.66 Mb, covering ∼99.35% of the estimated genome size. Compared with the genome of *V. vinifera* (87.65%) and *V. amurensis* (82.62%), the proportion of chromosomes in the genome of *V. zhejiang-adstricta* (88.44%) is higher. The BUSCO and LAI parameter validating the genome of *V. zhejiang-adstricta* is a reference genome. We annotated 31,196 genes and found more repetitive elements (especially intact LTR retrotransposons) than in the genomes of *V. vinifera* (PN40024). Synteny analysis of *V. zhejiang-adstricta, V. vinifera* and *V. amurensis* showed high collinearity between the three genomes, and few chromosome-level translocations were found between the three of the *Vitis* genome (**Fig. 3C**). Alignment and correction of Nanopore long reads confirmed that the *V. vinifera* and *V. amurensis* gene order in the primary assembly was correct, suggesting that the rearrangements between *V. zhejiang-adstricta* are real and not an assembly artifact. In addition, the rapid expansion of the most intact LTR may have affected the increased genome size of *V. zhejiang-adstricta*, and the expanded peaks of LTR appeared independent of *V. vinifera*. This genome shows high synteny with the previously published version but with higher coverage and more protein-coding gene sequences.

In contrast to the previously published genome of PN40024 (Jaillon et al. 2007) and *V. amurensis* (Wang et al. 2021), *V. zhejiang-adstricta* is a rare and endangered wild grape endemic to China, which survives within a narrow geographical region and lacks effective protection. There are relatively few studies on this species on account of the lack of genome information at present. However, previous studies have indicated that *V. zhejiang-adstricta* has a certain degree of disease resistance, and our study has indicated that it has a close relationship with *V. vinifera* and can be used as a parent donor for disease resistance breeding. This genome provides a data foundation and genomic guarantee for studying the environmental adaptability and disease resistance of *V. zhejiang-adstricta*. Further, it has great significance for protecting *V. zhejiang-adstricta* resources at the genomic level and reporting the diversity of grape germplasm resources. This high-quality assembled genome provides an important resource for evolutionary and functional studies in wild grape resources, especially given the importance of *V. zhejiang-adstricta* as a key protected wild, rare and endangered ancient grape.

### *V. zhejiang-adstricta* is an ancient Chinese unique wild grape

Most *Vitis* species indigenous to East Asia are wild grapes, different from *V. vinifera*, which has the highest species diversity of the genus. The distribution of wild East Asian species spans from the frigid zone to the tropics, with extremely rich germplasm resources. Although these wild species are rarely tasted by humans, they have economic significance as a source of breeding for excellent traits, such as resistance. As a wild type material of East Asian grape, *V. zhejiang-adstricta* is mainly distributed in subtropical areas with high temperature and humidity environments and has good disease resistance. In this study, *V. zhejiang-adstricta* was at a relatively early position in the evolution of *Vitis. Vitis* is considered to be one of the slowest evolving species, representing a more conservative ancestral structure of the genome, which can be used to unravel the evolution and genome replication history of other eudicots. The phylogenetic analysis illustrated that WGT or polyploidization seems to have occurred during the evolutionary histories of *Vitis*, such as the WGT-γ event that occurred ∼122–164 Mya and is shared by all eudicots in the Jurassic period. Middle Miocene climate optimum (MMCO) is the closest climate suitability stage not affected by human activities, and the species of *Vitis* underwent rapid differentiation in the MMCO, during which the period of separation of *V. zhejiang-adstricta* from *Vitis* occurred. Then, the speciation of *V. zhejiang-adstricta* and the large-scale insertion of LTR events occurred in the late Miocene cooling stage (Nie et al. 2023). Considering these different niches, it shows the possible evolutionary history of diversity. Several factors, including TEs and WGD, have been proposed to account for variations in genome size. According to the Ks analysis of *Vitis*, the evolution time of *V. zhejiang-adstricta* indicates that it might be determined by its special geographical environment and species evolution rate. *V. zhejiang-adstricta* evolved slowly after speciation and retained the original characteristics of the species.

### The special environmental adaptability of *V. zhejiang-adstricta*

After the origin of *V. zhejiang-adstricta*, more frequent chromosome rearrangements may have occured. The recent segment duplication may further provide new insight into the disease resistance characteristics of *V. zhejiang-adstricta*. Expanded and contracted gene families reflect the evolution of a gene family as a random birth and death process and also demonstrate model gene gain and loss along each lineage of a phylogenetic tree. The high disease resistance and low biological growth are the specific characteristics of *V. zhejiang-adstricta*, and the reasons for the natural multiplication of its population but endangered. According to previous research, *V. zhejiang-adstricta* has good growth conditions and extensive disease resistance in humid and hot environments. In our study, we revealed that genes involved in WGD events were expanded in the genome of *V. zhejiang-adstricta*, and many of these genes highly correlated with disease resistance. In addition, the TF and R genes, expanded in the evolution of *V. zhejiang-adstricta*, were identified to be highly correlated with disease resistance. Furthermore, the expanded genes of *V. zhejiang-adstricta* are mainly used for stress response, and contracted genes were related to plant growth and development and primary metabolism. In combination with the WGD events and chromosome SV, we identified that the NBS-LRR gene family plays a pivotal role in accelerating germination and response to pathogens. In the independent evolution of *V. zhejiang-adstricta*, more stress response and stress resistance genes were retained and growth and development-related genes were lost. With the accumulation of harmful mutations during the evolution process, *V. zhejiang-adstricta* has a small plant and weak growth potential, while the narrow habitat also makes it vulnerable to extinction. But *V. zhejiang-adstricta* was preserved due to the expansion of the gene family of favorable disease resistance and environmental adaptability.

In summary, our study highlights the evolutionary history of the *V. zhejiang-adstricta* genome, providing a perspective for *V. zhejiang-adstricta* as a study for disease resistance of species-specific WGD events and subsequent subgenomic differentiation in plants. Phylogenetic analysis based on complete *V. zhejiang-adstricta* genomes showed clear boundaries separating them from *Vitis* and other plants. In the independent evolution of *V. zhejiang-adstricta*, more stress response and stress resistance genes were retained and growth and development related genes were lost. Our findings have improved the current understanding of various types of gene replication and have determined the origin and specificities of *V. zhejiang-adstricta*. Those specific genes play an important role in the biosynthesis of plant secondary metabolites and lays a stable foundation for further improving grape disease resistance breeding.

## Materials and Methods

### Plant material, DNA extraction and genome sequencing

*V. zhejiang-adstricta* (accession number IB-VB-01) was collected from JiuFu mountain natural reserve (118°93′11″E, 30°9′62″N), Lin’an District, Zhejiang province, China, in 2019 and now planted in the germplasm resource repositories of China National Botanical Garden. The plant materials for genome sequencing were all collected from the same individual (Jiang et al. 2015) (**Fig. 1A**).

The genomic DNA of *V. zhejiang-adstricta* was extracted using a DNeasy Plant Mini Kit following the cetyl trimethyl ammonium bromide protocol. The paired-end libraries (500 bp) were prepared according to Illumina’s standard protocol. At least 5 μg of genomic DNA was used for the construction of the small-insert paired-end library, and the libraries were sequenced using the Illumina Novaseq 6000 platform with the model of PE150 (Champaign-Urbana, IL, USA).

The long-reads cDNA library was prepared according to the Oxford Nanopore Technologies MinION standard protocol using the SQK-LSK109. The obtained high-quality DNA sequences, the Nanodrop and Qubit were used for detecting nucleic acid, and 0.35% agarose gel electrophoresis was used for testing the purity, concentration and integrity of DNA. A BluePippin reagent kit was used to select the large fragment of DNA and was subjected to gelatinization recovery by applying the BluePippin automatic nucleic acid recovery instrument. The DNA library was sequenced on the Nanopore platform (van Dijk et al. 2018).

### Reads reconciliation and genome survey of *V. zhejiang-adstricta*

The Illumina reads were filtered by removing adapter contaminations, PCR duplicates and sequencing errors. Briefly, the main steps of data filtering using Trimmomatic (Bolger et al. 2014) are as follows: (i) trimming the adapter by aligning the reads to the adapter sequence, (ii) the reads from a short insert-size library if they contain more than 10% of unidentified bases (Ns) separately, (iii) removing the reads whose lengths were lower than 90 bp and (iv) aligning the sequencing reads and only keeping the paired reads.

The genome size of *V. zhejiang-adstricta* was estimated by k-mer analysis of Illumina sequencing data using Jellyfish (Marçais and Kingsford 2011). Based on the ‘k-mer’ depth distribution, the genome size was estimated according to the main peak *V. zhejiang-adstricta* and ‘K-mer’ number. Genomescope (Vurture et al. 2017) was used to predict the heterozygosity of *V. zhejiang-adstricta*.

### The genome assemble strategy

*V. zhejiang-adstricta* Nanopore long-reads data were first corrected using CANU (Koren et al. 2017) v1.7 with default parameters. Next, the high-quality data were used as input to wtdbg2 (Ruan and Li 2020) for a preliminary assembly with the mode of ccs, and the contig file was obtained for this assembly. Then, the Nanopore raw data and Illumina data were mapped onto the preliminary assembled contigs using Minimap2 and BWA, respectively. The assembled contigs were polished three times, and the polished scaffolds were eventually generated using Pilon (Walker et al. 2014). The gaps were closed after the construction of scaffolds using GapCloser. Purge Haplotigs (Roach et al. 2018b) were used for auxiliary processing of the high-heterozygosity scaffolds, evaluating the proper assignment of contigs and remaining one of them to the Haplotig.

The Hi-C reads of wild relative *V. adstricta* were used to generate pseudo-chromosomes. The mapped Hi-C reads to the assembled genome were assessed using Juicer (Durand et al. 2016b), and the non-duplicate mapped results were used as the input files of 3d-DNA (Dudchenko et al. 2017) to obtain the chromosome-level genome. With the parameters of three rounds (-r 3), the high-quality genome of the *V. zhejiang-adstricta* genome was assembled and the interaction and visualization of assembled genomes were drawn using Juicebox (Durand et al. 2016a).

### Assessment of the genome assembly and annotation

To evaluate the accuracy and integrality of the *V. zhejiang-adstricta* assembly, three methods were used. First, the WGS datasets were mapped to the genome, the mapping rate was measured using BamTools and the error rate was obtained by using the in-house Perl script. Then, we ran BUSCO using a total of 2,326 orthologous groups from plant lineages. We also processed an evaluation by LAI using LTR_retriever (Ou et al. 2018).

### Genome annotation

The *V. zhejiang-adstricta* repeats library was constructed by using RepeatModeler (Flynn et al. 2020) (version 1.0.11). The repeat sequences were identified using RepeatMasker (Chen 2004) (version 4.0.7) against the built libraries.

The prediction of protein-coding genes was *V. zhejiang-adstricta* based on ab initio gene prediction, homology-based gene prediction and RNA-Seq-aided gene prediction. The RNA-Seq dataset from four representative tissues was assembled with or without the reference genome by TRINITY (Grabherr et al. 2011), and then the assembled result was used to predict the gene model by PASA (Haas et al. 2008a). *Ab initio* gene models used for each gene predictor were trained from a set of high-quality candidate genes obtained from the PASA results. The *ab initio* gene models were trained and predicted using Augustus (Stanke and Morgenstern 2005), SNAP (Korf 2004) and GeneMark.hmm (Lukashin and Borodovsky 1998) with default parameters. In homology-based gene prediction, GenomeThreader (Gremme et al. 2005) was used to align the protein sequences from other *Vitis* species (PN40024, *Vitis amurensis*, etc.) to the assembly genome and determine gene structures. Finally, the EvidenceModeler (Haas et al. 2008a) was used to integrate all these annotation files.

The rRNAs based on the basic Eukaryotic rRNA database were predicted using a homology-based method with BAsic Rapid Ribosomal RNA Predictor search by using an *e*-value of 1e-5. The tRNA information in the *V. zhejiang-adstricta* genome was identified by tRNAscani-SE (Lowe and Eddy 1997) (version 2.0.7) using the default settings. Other noncoding RNA annotations were determined using Infernal (Nawrocki and Eddy 2013) (version 1.1.2) with the default parameters.

### Gene function annotation and resistance (R) gene identification and comparative genomics analysis

Gene function was inferred according to three functional databases, including NCBI non-redundant Nr (Deng et al. 2006), Pfam (Mistry et al. 2021) and Uniprot (Consortium 2018) databases; BLAST was used to search the similar hits of each protein (*e*-value 1e-5), and only the best hits were kept. Gene Ontology (The Gene Ontology Consortium 2017) and KEGG (Kanehisa et al. 2017) IDs for each gene were obtained from eggnog-mapper (Huerta-Cepas et al. 2017).

To identify the R gene in the *V. zhejiang-adstricta* genome, HMMER software was used to scan the predicted R gene with the PF00931 hidden Markov model (HMM) constructed according to the NB-ARC domain in the Pfam database and known reference, and putative R-genes belonging to 192 plant species were downloaded from the PRGdb database (Calle García et al. 2022). R-Genes can be functionally grouped into at least six distinct classes based on the presence of specific classes: CNL, TNL, RLP, RLK and other classes. R-Genes can also be functionally grouped into at least six domain types: CC, kinase, LRR, NBS, TIR and TM. The pivotal domain for the predicted R genes was indicated by drago and determined by the NCBI Conserved Domain Database (CDD) and Pfam database (Mistry *et al*. 2021).

### SV identification and MCScan analysis of *V. vinifera* and *V. amurensis*

The SMRT PacBio data of *V. vinifera* were downloaded from the NCBI database (Velt et al. 2023), and the PacBio data of *V. amurensi* were collected from the preliminary work and sequencing in the laboratory (Wang et al. 2021). The PacBio datasets of *V. vinifera* and *V. amurensi* were used to detect the SV between the genome of *V. zhejiang-adstricta*. The PacBio datasets were first corrected by CANU and then aligned by Minimap2 (Li 2018). The alignment bam was sorted using SAMtools, and the SVs were identified using cuteSV (Jiang et al. 2020) with the parameters of –max_cluster_bias_INS 100, –diff_ratio_merging_INS 0.3, –max_cluster_bias_DEL 100 and –diff_ratio_merging_DEL 0.3 -s 3 -r 1000. The pairwise synteny visualization of *V. zhejiang-adstricta* and *V. vinifera* or *V. amurensis* was constructed using MCScan.

### Phylogenetic analysis and gene family expansion in plants

In addition to *V. zhejiang-adstricta* and the other 14 plants [*A. thaliana* (Meinke et al. 1998), *O. sativa* L. (Yu et al. 2002), *C. papaya* (Ming et al. 2008), *Actinidia chinensis* Planch. (Yue and Shi 2021), *Lycopersicon esculentum* (Sato et al. 2012)*, Theobroma cacao* L. (Argout et al. 2011), *Citrus sinensis* (Xu et al. 2013), *C. rotundifolia* (Xin et al. 2022), *V. vinifera* (Jaillon et al. 2007), *Populus trichocarpa* (Tuskan et al. 2006), *Amygdalus persica* L. (Du et al. 2021), *Malus domestica* (Velasco et al. 2010), *Fragaria × ananassa* (Edger et al. 2019) and *Coffea arabica* (Tran et al. 2018)], protein sequences were collected for the constructed phylogenetic relationship. OrthoMCL (Li and L 2003) (version 1.1.4) was used to identify orthologous groups. First, compliantFasta was used to filter the protein sequences with the threshold of sequence length greater than 30 bp and with 20% proportion of stop codons. Then, all-vs-all BLASTP was used to align all protein sequences for 15 plants with an *e*-value cutoff of 1e-05. Paralogous and orthologous genes were clustered by OrthoMCL. In the phylogenetic analyses, single-copy genes were determined from clustered genes and the selection of the single-copy genes was aligned for multisequence comparison using mafft (version 7.305b). The sequences after alignment for each family were them trimmed by Trimmal, then all muti-fasta files were combined into a super alignment matrix and the combined file was used to generate a phylogenetic tree by RaxML (Stamatakis 2014) (version 8.2.9) with the model of ‘PROTGAMM+JTT+X’ and a bootstrap of 1,000.

The MCMCtree program (version 4.8a) implemented in PAML software (Yang 2007) was used to estimate the divergence time. The median and adjusted times of *A. thaliana*, *O. sativa* L. and *C. papaya* were derived based on the divergence time from TimeTree (Kumar et al. 2017). The expansion of gene families was processed using CAFÉ (Bie et al. 2006) (version 3.1). Chromosome rearrangements in *V. zhejiang-adstricta* were investigated using AEK genes, and the software MCscanX was employed to identify syntenic blocks shared between the AEK and the aimed species.

### Phylogenetic analysis and insertion time analysis of LTR-RTs in *Vitis*

The phylogenetic analysis was inferred with eight *Vitis* species (*C. rotundifolia, V. vinifera, Vitis sylvestris, V. amurensis*, *V. arizonica*, *V. riparia*, *M. rotundifolia* and *V. zhejiang-adstricta*) protein sequences using a similar method to plant phylogenetic analysis; 41 Chinese wild *Vitis* accessions were obtained from the *Vitis* germplasm from a previous study (Liang et al. 2019), and the BioProject accession is PRJNA393611. Paired-end resequencing reads were aligned to the *V. vinifera* reference genome by BWA using the default parameters, and SAMtools was used to convert mapping results into the BAM format. The Genome Analysis Toolkit was used for variation detection. After the filtering step, the variations were filtered with the parameter of none bi-allelic, >99% missing calls and MAF < 0.05. We used the high-quality SNPs to construct the phylogenetic tree of Chinese wild *Vitis* accessions with 100 bootstraps using FastTree (Price et al. 2009). The growth potential analysis of grapes was performed through *Vitis* evolutionary tree nodes containing *V. zhejiang-adstricta, V. vinifera* and 10 other wild grapes. To conduct the statistical analysis of internode length and width, leaf length and width, and petiole length in new shoots, the growth potential of *V. zhejiang-adstricta* was analyzed.

All-vs-all BLASTP was employed between *V. zhejiang-adstricta* and *V. vinifera* with an *e*-value of 1e-05. Then, MCScanX was used to identify the synteny blocks with at least five genes in each pair. The Ka and Ks values were calculated with KaKs_Calculator (Wang et al. 2010) (v2.0) using the NG estimation method. The LTR-RT analysis was conducted to determine the insertion time of LTR-RTs in *Vitis* species. These LTR-RTs were aligned with mafft, and the nucleotide divergence rate (λ) is 5.4e-9 (Liang et al. 2019). LTR_retriever was employed to estimate the insertion time of all intact LTR-RTs in *Vitis*.

## Supplementary Material

pcad140_Supp

## Data Availability

The public software used in this work is cited in the Materials and Methods section. If no detailed parameters were mentioned for the software, default parameters were applied with guidance. The *V. zhejiang-adstricta* genome assembly project has been deposited at the National Genomics Data Center (NGDC) under BioProject number PRJCA007751. The Illumina and Nanopore data of *V. zhejiang-adstricta* have been deposited under Biosample accession SAMC3129443 and SAMC3129445. The Hi-C data of *V. adstricta* have been deposited under Biosample accession SAMC3129444. The improved gene prediction, coding and peptide sequence repeat information and other detailed information of this genome can also be accessed from the newly developed Grapeworld platform (http://www.grapeworld.cn/zjyy/download.html).
