# Supplementary material for: The Genome of Vitis zhejiang-adstricta Strengthens the Protection and Utilization of the Endangered Ancient Grape Endemic to China
Source: Plant Cell Physiol. 2023 Nov 1;65(2):216–27. doi: 10.1093/pcp/pcad140 (PMC10873524; doi:10.1093/pcp/pcad140)
Supplement: pcad140_Supp [file pcad140_supp.zip › suppl_data/pcp-2023-e-00137-File007.docx]

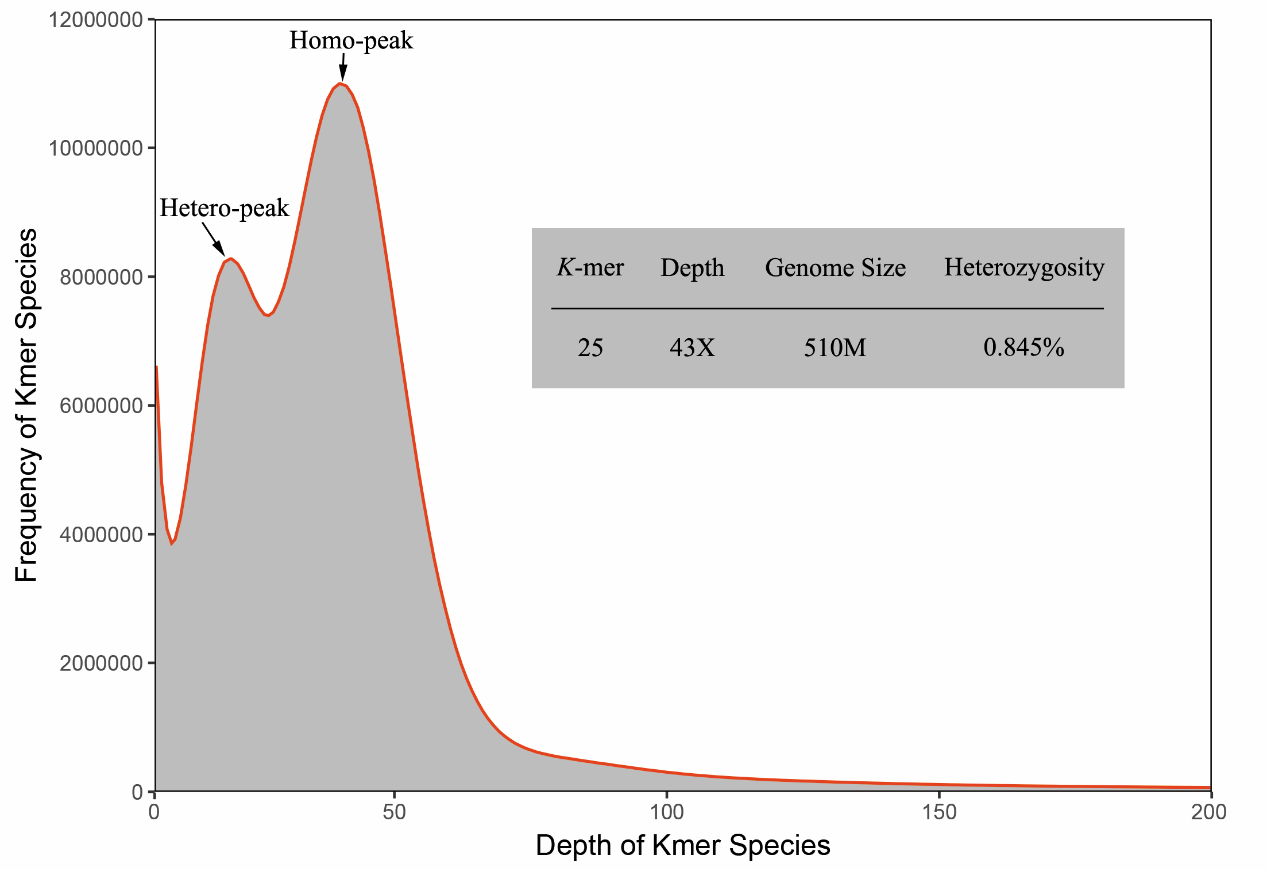


Figure S1. K-mer analysis of *V. zhejiang-adstricta*. The used k-mer is 25, the depth of hetero peak is 20, and homo-peak is 40. The depth of sequencing data is 43x, and 25-mer frequency distribution of sequencing reads estimates the genome size of *V. zhejiang-adstricta* is 510M and the heterozygosity of *V. zhejiang-adstricta* is 0.845%.


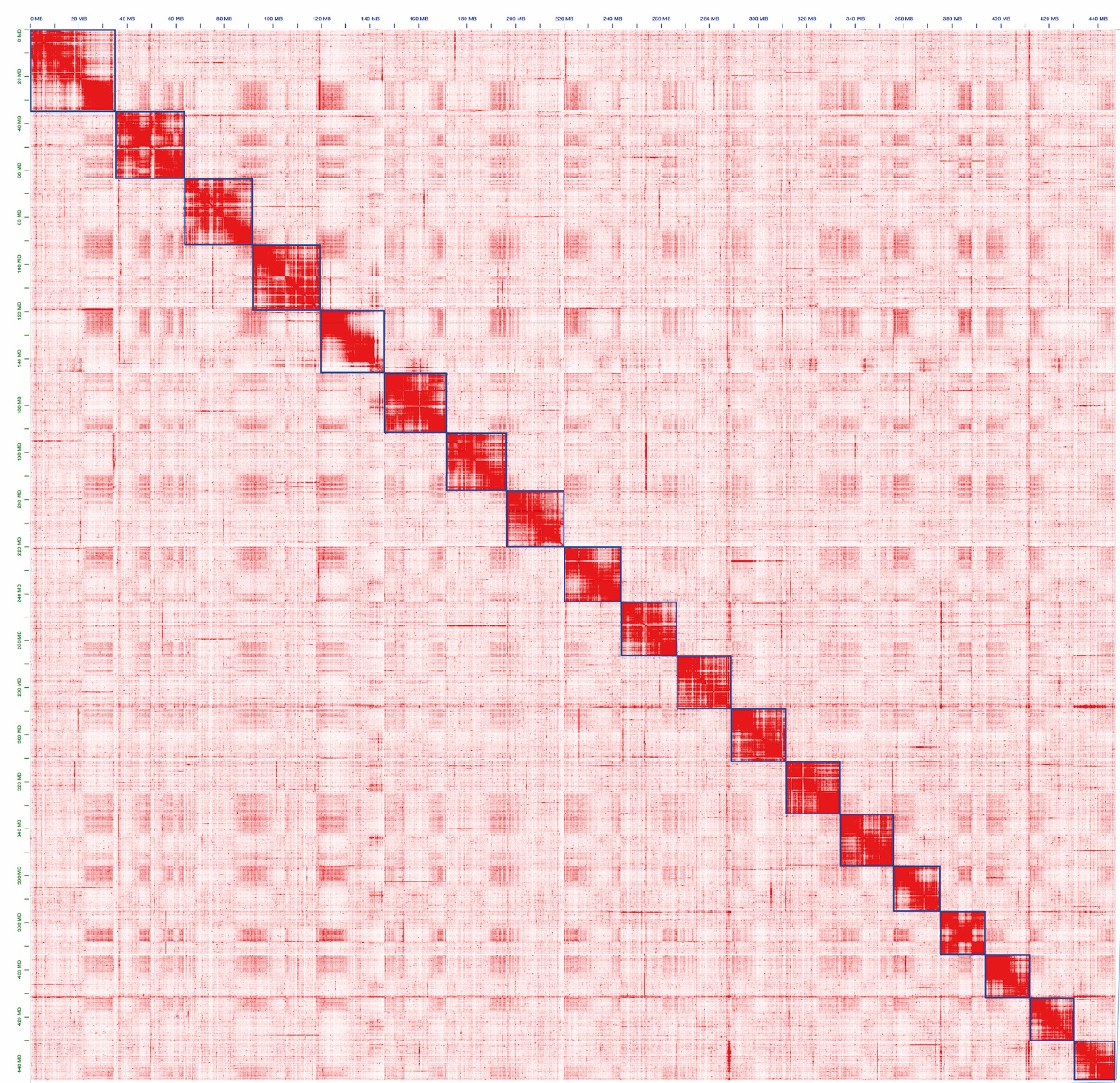


Figure S2. Hi-C chromatin interaction heatmap for the 19 pseudochromosomes of the *V. zhejiang-adstricta* genome. The x-axis and y-axis stand for the length of assembled genome, the blue blocks indicate the chromosome boundary, the color depth implies the interact intensity.


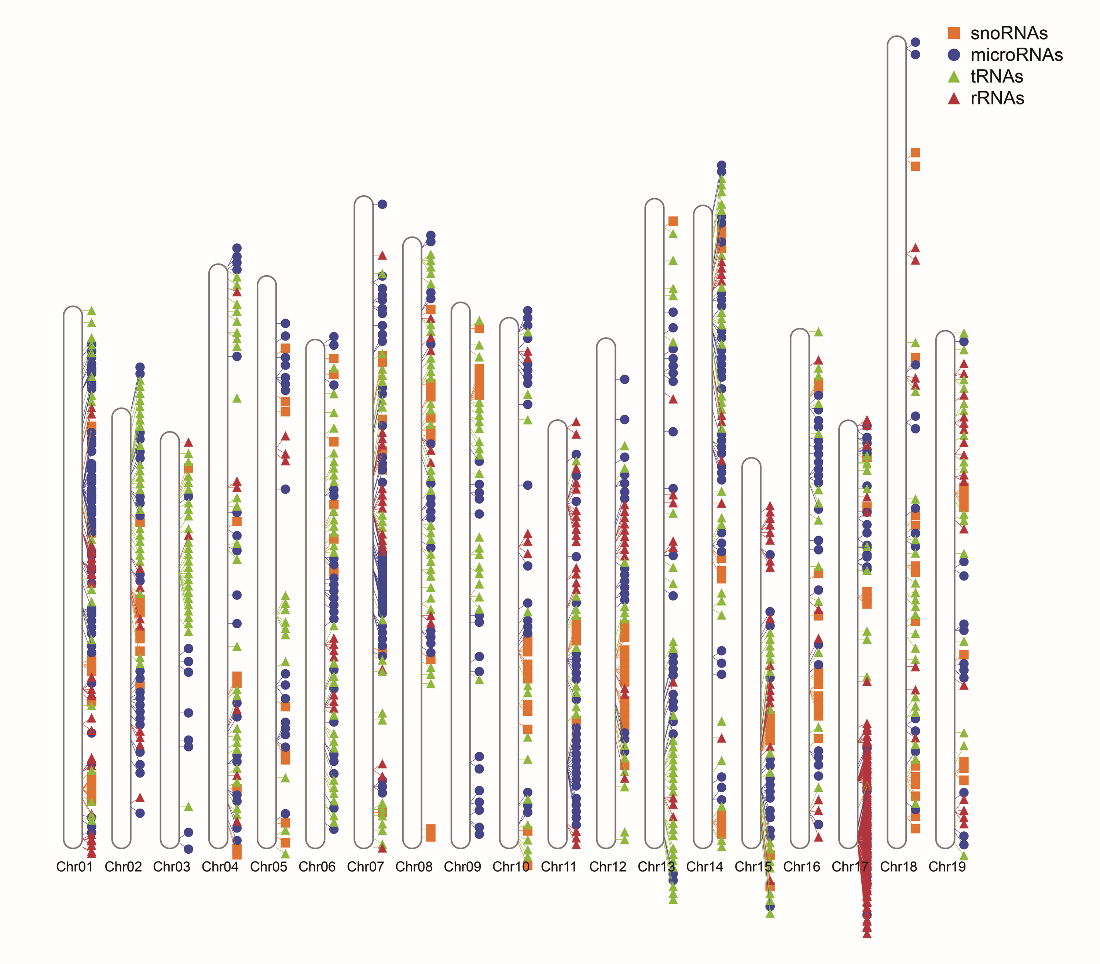


Figure S3. Distribution of four type small RNAs in *V. zhejiang-adstricta* genome. The heatmap on the chromosome indicated the low and high small RNAs density of each area.


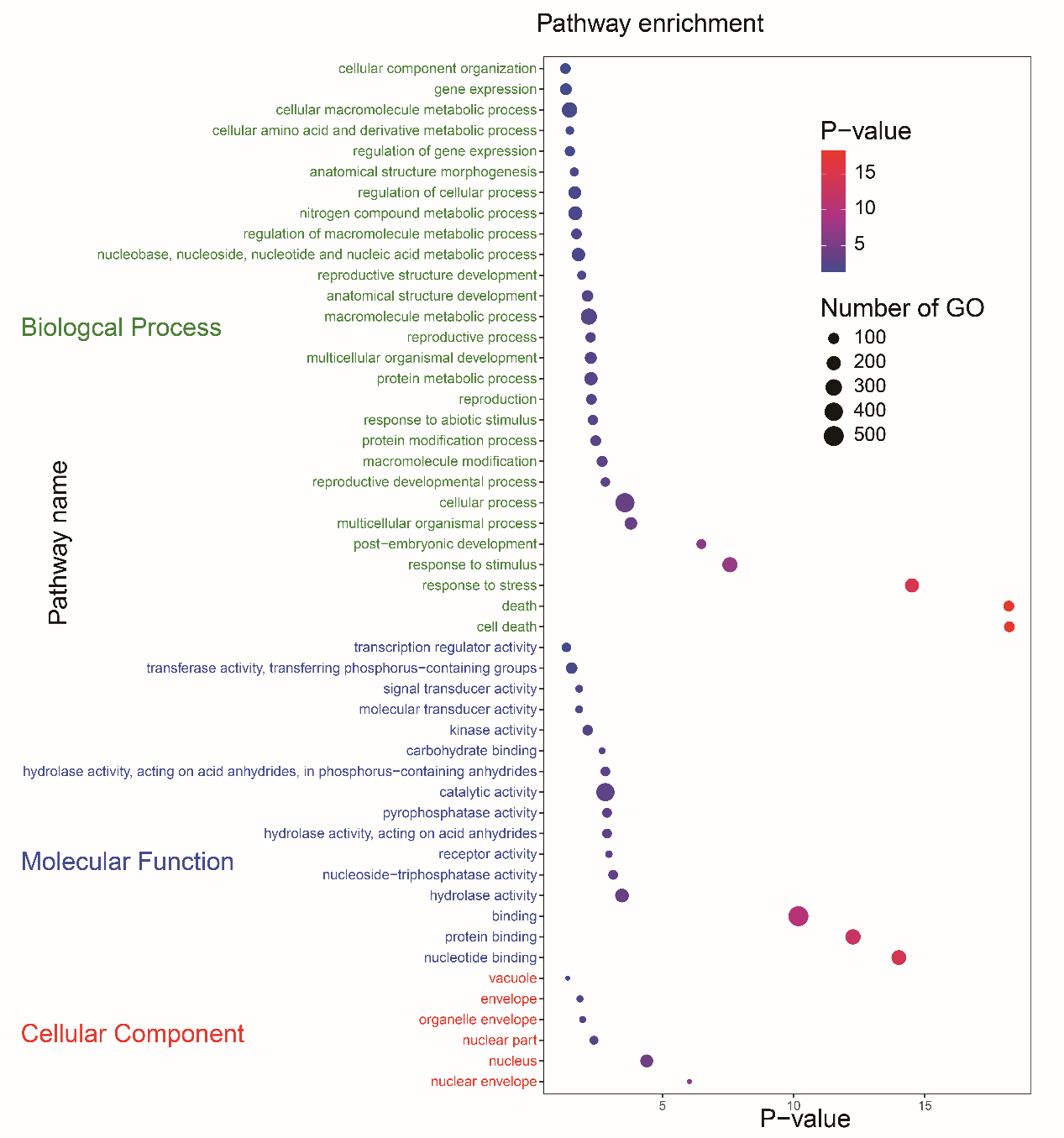


Figure S4. GO enriched analysis of the unique genes in *V. zhejiang-adstricta* genome, the input is unique genes and the background is the whole gene of the assembled genome.

**
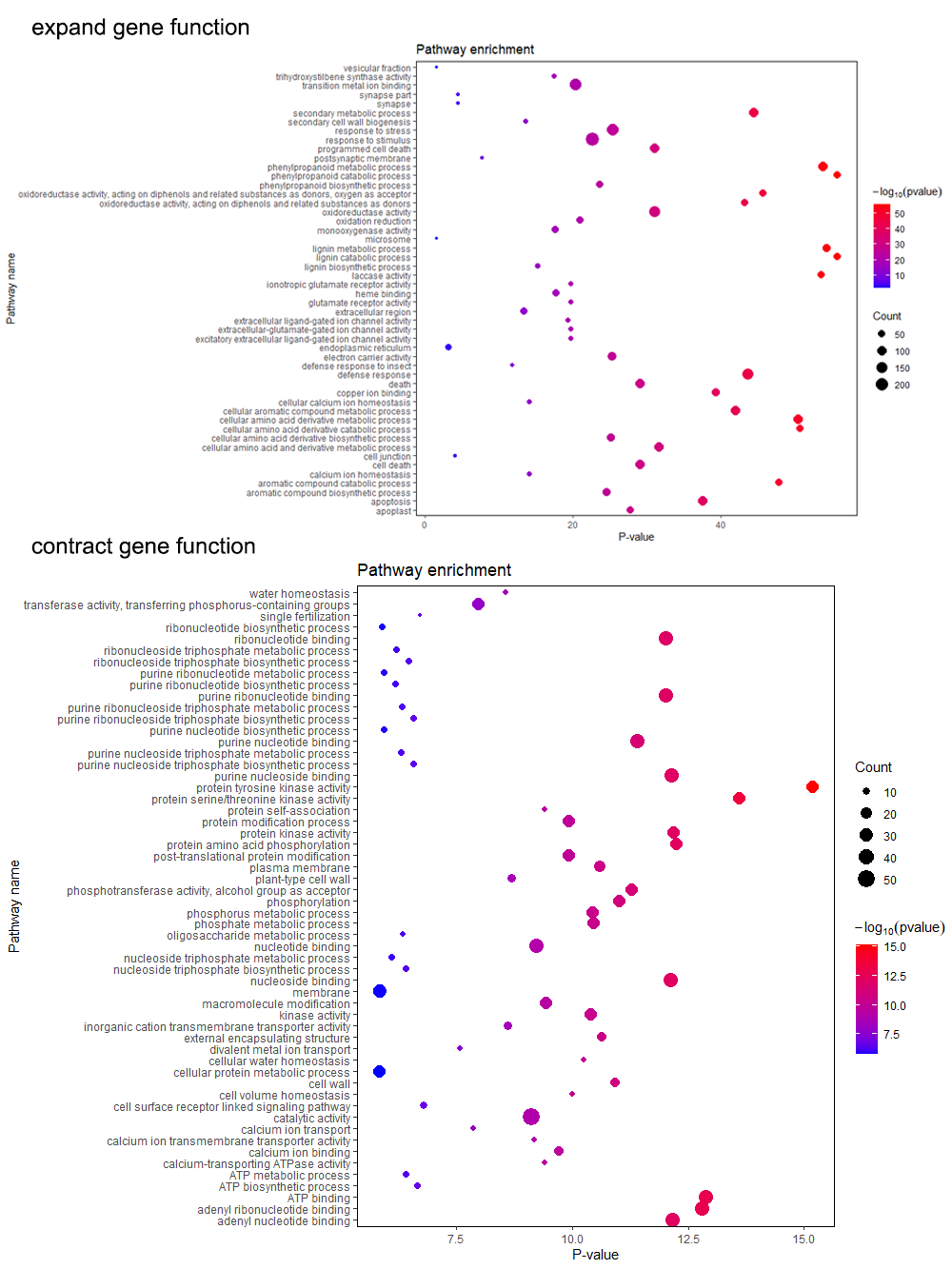
**

Figure S5. GO enriched analysis of the expanded and constracted gene families in *V. zhejiang-adstricta* genome, the input is the expanded and constracted gene families and the background is the whole gene of the assembled genome.


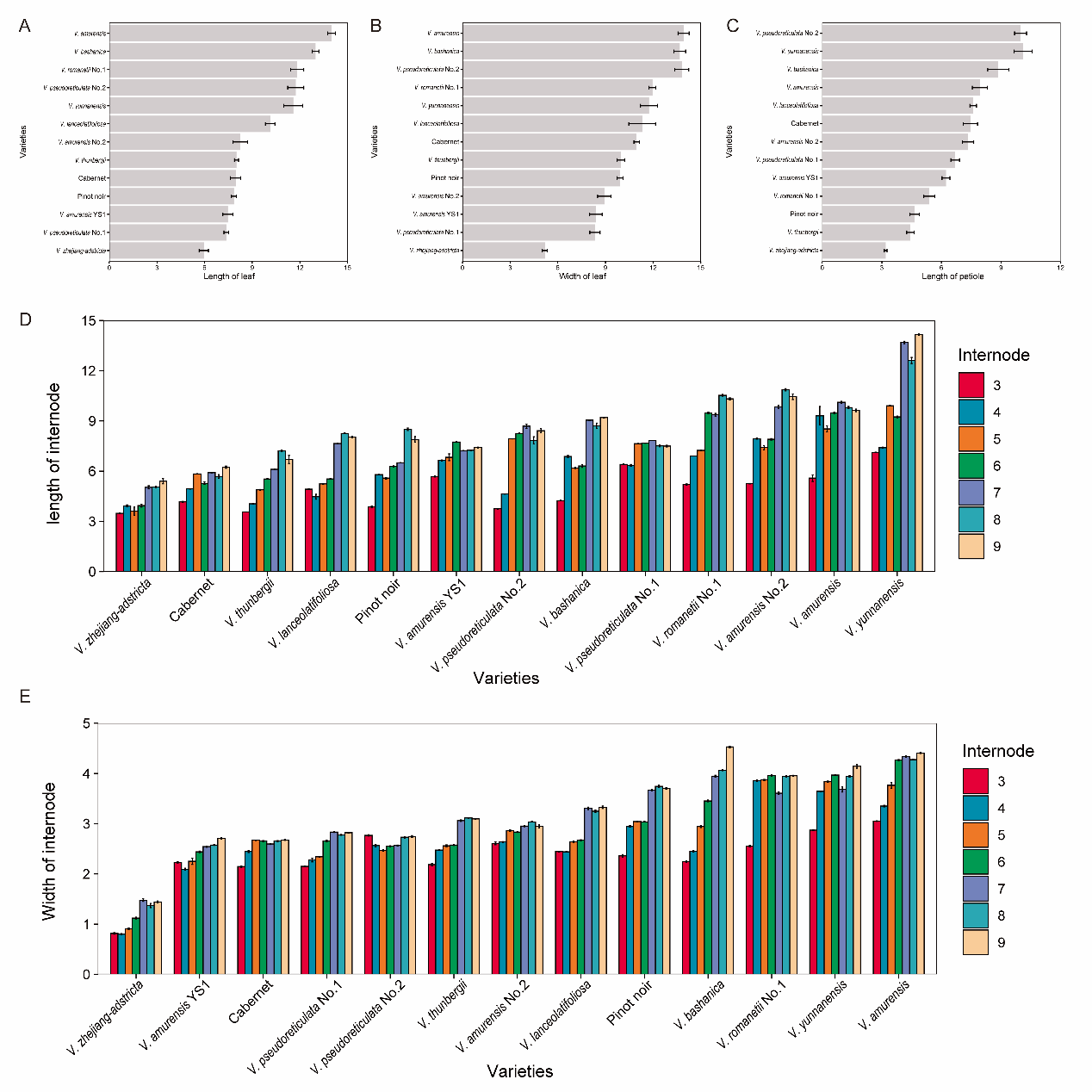


Figure S6. The growth trend analysis of wild grapes in China. A. The length of leaf of 11 Chinese wild grapes and 2 *V. vinifera*. B. The width of leaf of 11 Chinese wild grapes and 2 *V. vinifera*. C. The width of petiole of 11 Chinese wild grapes and 2 *V. vinifera*. D. The length of internode of 11 Chinese wild grapes and 2 *V. vinifera*. E. The width of internode of 11 Chinese wild grapes and 2 *V. vinifera*.


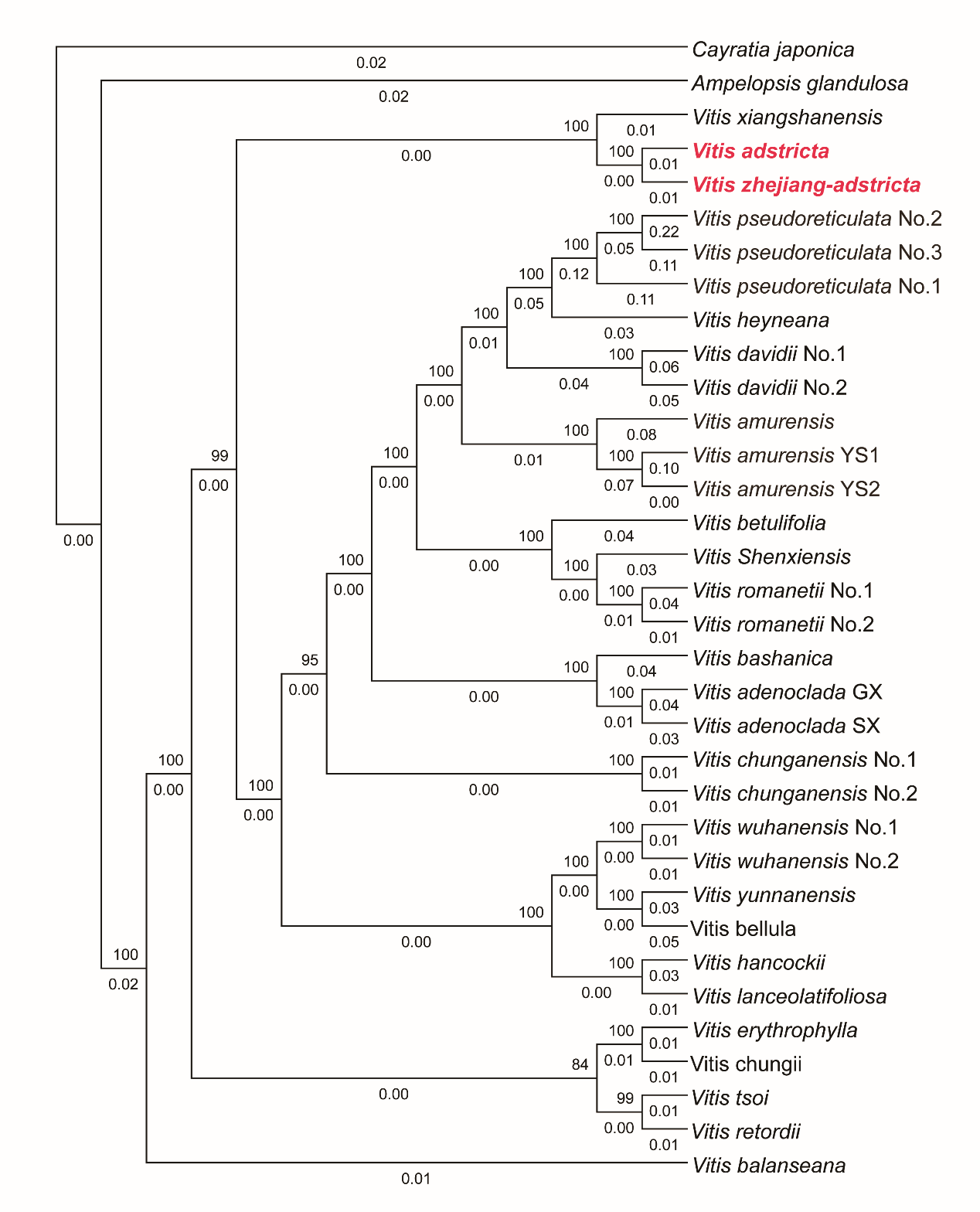


Figure S7. Evolution and comparative analysis of chinese wild grape. The *V. zhejiang-adstricta*, close-related species *V. bryoniifolia*, *Vitis pseudoreticulata* and *V. amurensis* are marked in different color.


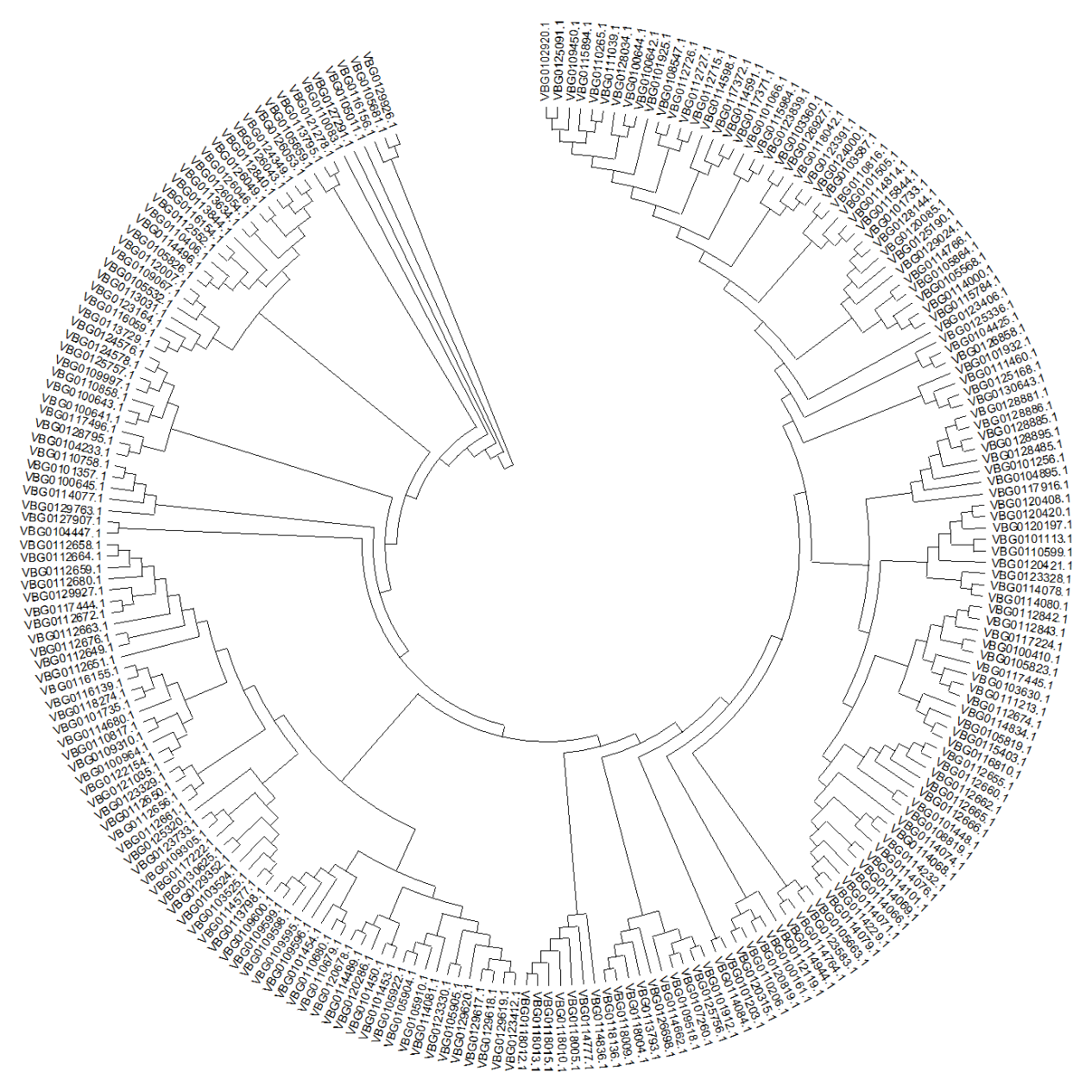


Figure S8. Evolution and comparative analysis of NBS-LRR gene family in *V. zhejiang-adstricta*.
